# Hydrogel In-Tape Electronic Tongue

**DOI:** 10.1021/acsaelm.4c02059

**Published:** 2025-02-26

**Authors:** Ricardo Brito-Pereira, Rita Policia, Clarisse Ribeiro, Pedro Martins, Senentxu Lanceros-Mendez, Frank N. Crespilho

**Affiliations:** † BCMaterials, Basque Center for Materials, Applications and Nanostructures, UPV/EHU Science Park, 48940 Leioa, Spain; ‡ CF-UM-UP, Centro de Física das Universidades do Minho e Porto, Universidade do Minho, Campus de Gualtar, 4710-057 Braga, Portugal; § IB-S, Institute of Science and Innovation for Bio-Sustainability, Universidade do Minho, Campus de Gualtar, 4710-057 Braga, Portugal; ∥ IKERBASQUE, Basque Foundation for Science, 48009 Bilbao, Spain; ⊥ São Carlos Institute of Chemistry, University of São Paulo, São Carlos 13560-970, SP, Brazil

**Keywords:** electronic tongue, sustainable sensor, hydrogel in-tape sensor, sequential analysis, beverage classification

## Abstract

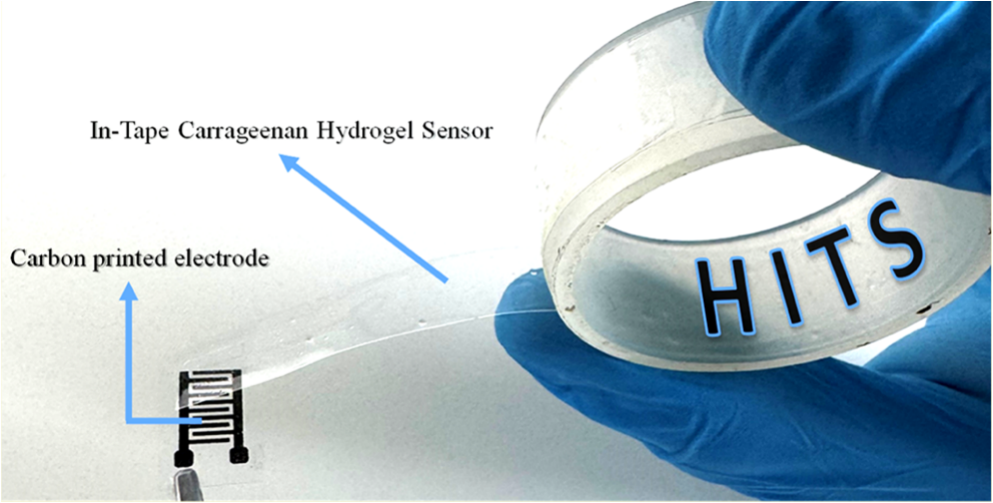

An electronic tongue is a sensor-based system designed to mimic human taste by detecting and analyzing the chemical properties of liquids through electrochemical methods. Here, we introduce the HITS concept, an electronic tongue system that enables rapid and sequential classification of various beverages. This system utilizes a single, cost-effective platform with interdigital electrodes made of carbon printed on recyclable poly(ethylene terephthalate) (PET), significantly reducing the need for multiple electrodes. With the use of interchangeable hydrogel tapes, the system requires a single deep (150 μL) pore per analysis, allowing for efficient sequential testing. The hydrogel seamlessly accommodates the electrode interface and operates with a semisolid electrolyte, achieving ultrafast analysis times of just 5 min. Employing AI and machine learning algorithms, HITS accurately differentiated between coffee, juice, water, white wine, and red wine with a 100% success rate. This sustainable approach combines high precision, speed, and low environmental impact, offering a versatile solution for various technological applications, including food science, quality control, and health monitoring. This e-solution not only enhances precision and speed but also aligns with growing environmental concerns, offering a low-impact and scalable platform for advanced liquid analysis.

## Introduction

More than two decades ago, *Nature* featured an editorial titled ″*Electronic tongue has good taste*,″ highlighting pioneering work on a sensor known as the electronic tongue (e-tongue), which could accurately distinguish the four basic tastes (salty, sour, sweet, and bitter) using devices made from ultrathin films deposited onto gold interdigitated electrodes.^1,2^ Nowadays, the e-tongue is becoming increasingly implemented across various industries, including food and beverage, pharmaceuticals, and environmental monitoring.^3^ Its ability to analyze the chemical properties of liquids makes it invaluable for applications such as quality control, product development, and counterfeit detection.^4,5^

However, one of the longstanding challenges in electronic e-tongue technology is the complexity and overlap of the electrochemical signals generated by nonspecific sensors, which makes it difficult to achieve reproducible and accurate results.^6,7^ Traditional methods often rely on complex data libraries and pattern recognition techniques that struggle to distinguish between subtle variations in taste profiles.^8,9^ These signals, such as changes in electrical impedance, potentiometric shifts, or voltammetric patterns, can be influenced by a variety of factors, including sensor drift, environmental changes, and cross-sensitivity to multiple analytes.^10^ One common approach is electrochemical impedance spectroscopy (EIS), which excels at probing the complex interfacial processes at the sensor surface, such as charge transfer, diffusion, and adsorption phenomena.^11^ EIS provides detailed information on the interactions between analytes and sensor surfaces by measuring impedance over a range of frequencies.^11^ However, its application in electronic tongues faces challenges, particularly in achieving consistent and interpretable results across varied samples. These difficulties stem from factors like sensor drift, the nonhomogeneous nature of liquid samples, and variations in environmental conditions (e.g., temperature, humidity, and pH).^12^ Furthermore, interpreting EIS data often requires sophisticated models to decouple overlapping processes, making real-time analysis difficult, once the nonlinearity and high dimensionality of impedance spectra complicate the use of simple algorithms.^13^ Therefore, ensuring reproducibility and accurate interpretation remains an ongoing issue, highlighting the need for more robust signal processing and sensor design innovations in e-tongue applications.^13^

The primary difficulties arise from the complex impedance spectra, which often exhibit overlapping time constants and poorly defined semicircles in Nyquist plots, making it challenging to extract meaningful parameters such as charge transfer resistance (Rct) and double-layer capacitance (Cdl).^14^ This issue is particularly problematic in beverage samples, where the interfacial processes can vary significantly.^15^ Therefore, advanced modeling and fitting techniques, such as complex nonlinear least-squares (CNLS) and equivalent circuit modeling, are required to interpret the results. Despite these techniques, the inherent variability in liquid samples can still lead to significant challenges in achieving reproducible and meaningful interpretations due to subtle differences in the composition or electrode surface conditions, which can strongly affect the impedance response, kinetic processes involving electron or ion transfer between the electrode and the analytes, and diffusion processes, represented by Warburg impedance (ZW).^16,17^ Another layer of complexity arises from the thermodynamic aspects, represented mainly by Cdl and energy barriers for charge transfer. In biological samples, the complexity is further amplified by varying analytes, proteins, ions, and biomolecules, which introduce overlapping kinetic signatures. This makes Nyquist plots more convoluted, especially when large biomolecules adsorb onto the electrode, altering Cdl and changing impedance behavior.^18,19^

Given these technical challenges, the integration of machine learning (ML) and artificial intelligence (AI) in e-tongue applications offers promising solutions. For example, ML algorithms, such as Random Forests and Support Vector Machines, and deep neural networks can be implemented to identify key features within the complex signal landscape, filtering out noise, and improving the specificity of detection. This approach reduces the dependence on prebuilt data libraries and enhances the system’s adaptability, enabling more reliable and precise signal interpretation in applications like flavor profiling, quality control in the food industry, and even detection of contaminants in environmental monitoring.^20,21^

Beyond the technical challenges, there are also pressing environmental concerns.^22^ The rapid proliferation of electronic devices and their short lifespan have led to an alarming increase in nonbiodegradable electronic waste (e-waste), which poses significant environmental and health risks due to the presence of heavy metals and persistent organic pollutants.^23^ Developing recyclable, reusable, and biodegradable materials for sensors is crucial to addressing these challenges.^24^ By combining high performance with reduced environmental impact, sensors made from materials that either decompose naturally or can be reused significantly mitigate the environmental footprint associated with traditional electronics.^25−27^ Hydrogels, specifically those derived from natural sources, have shown great promise as alternatives for more sustainable sensor designs.^28^ Among these, iota-carrageenan, a natural polysaccharide derived from red seaweed, stands out due to its ability to form hydrogels that are biocompatible, nontoxic, and renewable.^29^ Carrageenan-based hydrogels are especially suited for sensor applications, as they retain substantial amounts of water, ensuring stable and accurate measurements. In sensor technology, this hydrogel’s ability to interact with analytes makes it particularly effective in real-time monitoring applications, such as food and beverage analysis.^30,31^

Here, we introduce an innovative, fully biodegradable electronic tongue (e-tongue) system called HITS (Hydrogel In-Tape Sensor), designed to enhance reproducibility, reduce variability, and provide rapid beverage analysis. The system integrates carbon interdigitated electrodes printed on recyclable poly(ethylene terephthalate) (PET) with a biodegradable iota-carrageenan hydrogel. This combination not only improves the stability and consistency of EIS measurements but also aligns with sustainable practices by using eco-friendly materials. HITS operates as a versatile platform that utilizes interchangeable hydrogel tapes, confining liquid samples uniformly within a gel electrolyte (Figure S1). This approach enables fast analysis times of 5 min per sample, requiring a single dip in the liquid sample for 3 min, for efficient sequential testing. By confining the sample to a single electrode, the system eliminates the need for multiple electrode arrays, significantly reducing the costs and electronic waste. The e-tongue was tested on various beverages, including coffee, juice, milk, red and white wine, and water, providing distinct chemical profiles. Impedance data collected across a frequency range of 10 Hz to 10 kHz were analyzed using principal component analysis (PCA), which successfully differentiated the beverages based on their unique chemical signatures influenced by pH level, ionic concentrations, and presence of organic compounds. ML and AI algorithms further enhanced the system’s performance, achieving a 100% success rate in classifying the beverages during blind sensory analysis.

## Results

**Figure 1:**
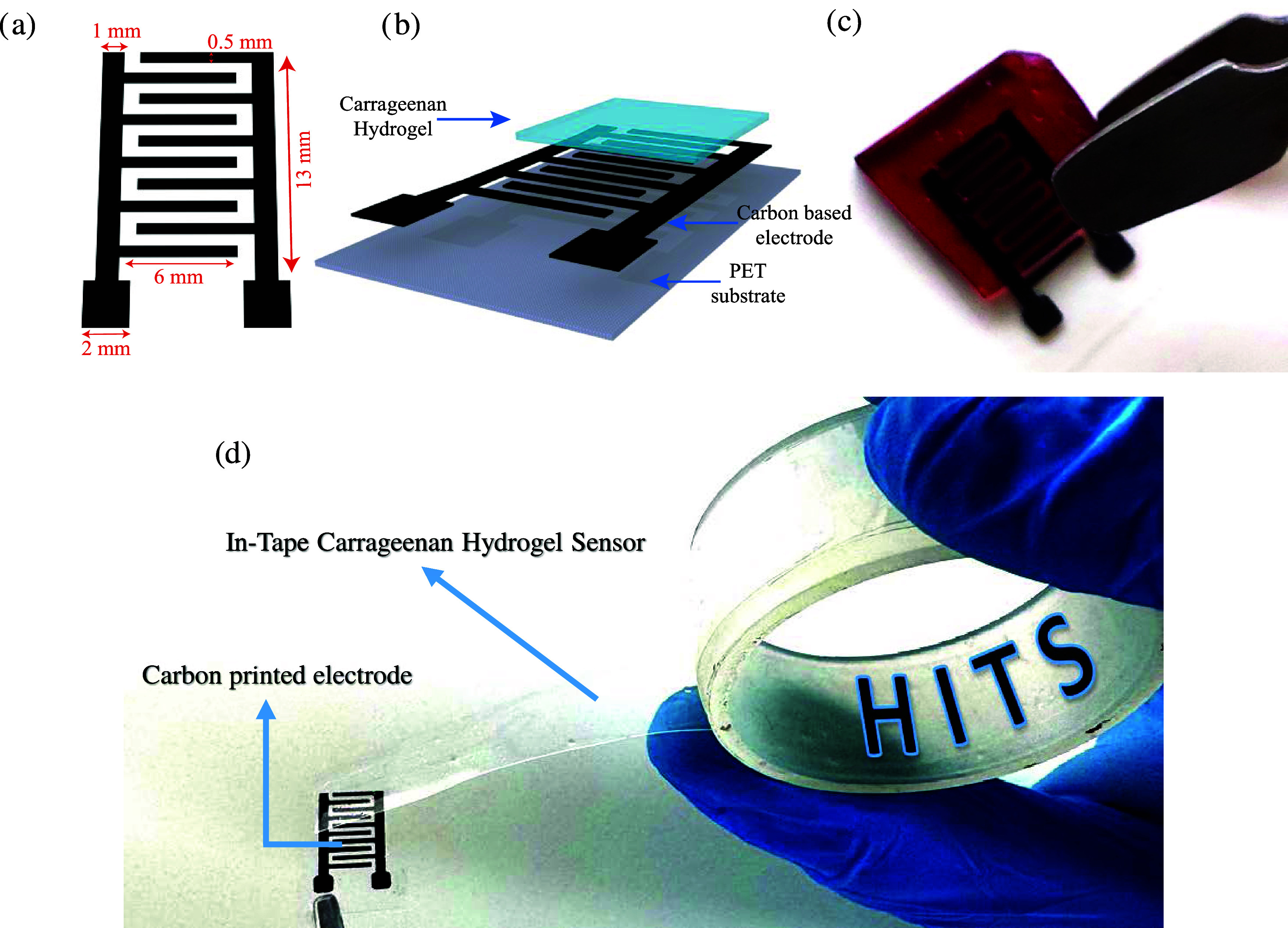
Design and assembly of hydrogel in-tape sensor (HITS) system. Schematic and photograph of the carbon-based interdigitated electrode on a recyclable PET substrate, integrated with a biodegradable iota-carrageenan hydrogel layer. (a) Carbon electrode dimensions, including line width, interdigit spacing, and overall size. (b) Layered structure of the sensor, showing the application of the hydrogel tape over the electrode. (c) Photograph of the fully assembled HITS sensor with the carrageenan hydrogel applied, ready for rapid and efficient beverage analysis. (d) Photographic picture illustrating the hydrogel in-tape sensor (HITS) concept. This configuration highlights the sustainable and versatile design, enabling ultrafast electrochemical measurements and minimizing e-waste.

The HITS system for beverage recognition was fabricated using a carbon interdigitated electrode coated with an iota-carrageenan hydrogel (Figure 1). The materials used included i-carrageenan, carbon black ink, PET substrates, and various beverages such as coffee, milk, red wine, white wine, and deionized water. The preparation involved fabricating the electrode, forming a hydrogel layer (Figure S1), and characterizing the sensor’s electrochemical and physical properties. Impedance measurements across different beverage samples were analyzed using ML techniques, including PCA and Random Forest algorithms, to classify the beverages based on their unique electrochemical signatures. All experimental details and data processing steps are provided in the Supporting Information (SI).

**Figure 2:**
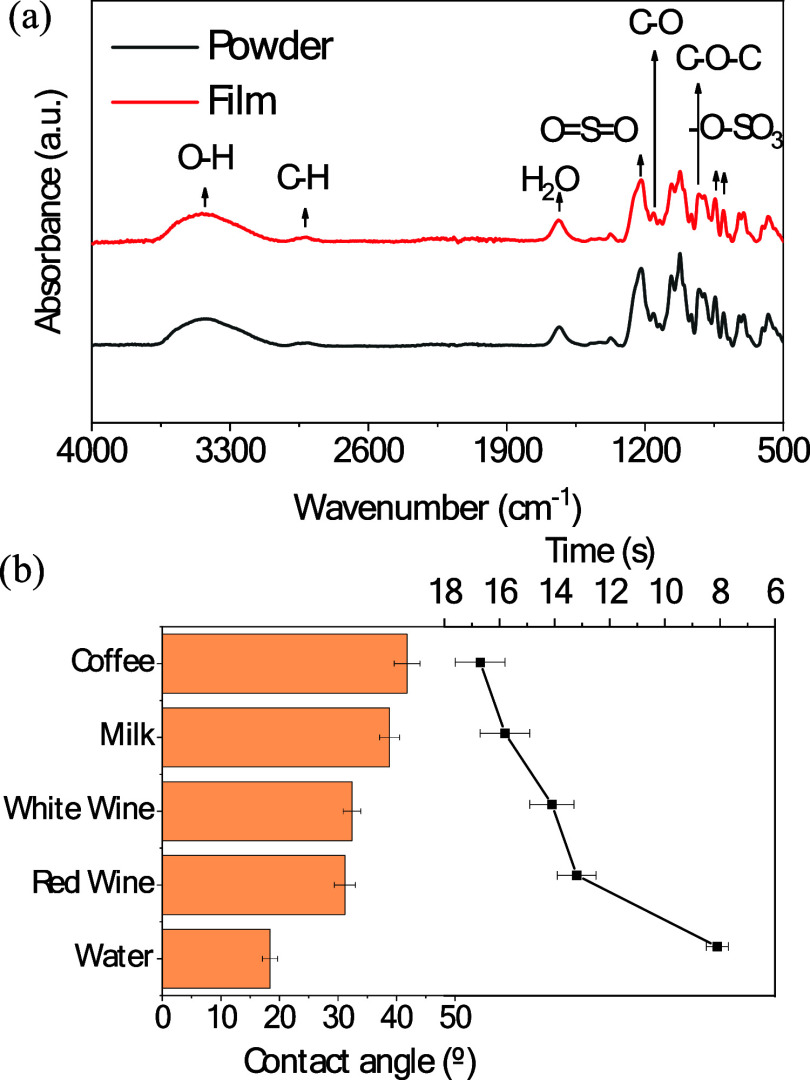
Characterization of i-carrageenan hydrogel in the HITS system. (a) FTIR spectra of the i-carrageenan hydrogel used in the HITS sensor, comparing its vibrational spectral characteristics in film and powder forms. The spectra highlight key functional groups that contribute to the hydrogel’s interaction with liquids during sensing. (b) Contact angle measurements for various beverages on the surface of the i-carrageenan hydrogel, demonstrating the wettability and absorption behavior critical for the HITS system’s performance. The duration for complete absorption of each liquid is also shown, providing insights into the hydrogel’s ability to uniformly confine samples for rapid and consistent electrochemical measurements.

The physicochemical characteristics of i-carrageenan were analyzed by using vibrational spectroscopy. A comparison of its spectra in powder and film forms was performed to determine whether the molecular structure of the polymer is preserved after hydrogel formation. FTIR spectra represented in Figure 2a present the typical i-carrageenan absorption bands at 845 and 802 cm^–1^, corresponding to the −O–SO_3_ stretching vibration at D-galactose-4-sulfate (G4S) and D-galactose-2-sulfate (DA2S), C–O bridge stretching at 1157 cm^–1^, and C–O stretching at 1066 cm^–1^.^32,33^ The lack of new absorption bands in the film spectrum indicates that the film processing method retained the molecular structure of i-carrageenan. However, the absorption bands corresponding to the O–H stretching vibration, C–H stretching vibration, and water deformation (adsorbed water) that appear in the powder sample at 3342, 2913, and 1636 cm^–1^, respectively, are shifted to lower wavelengths in the hydrogel spectrum, to 3420, 2906, and 1634 cm^–1^, respectively.^34^ This shift is probably due to hydrogen bonding formation between the water molecules and the carrageenan polymer chains.^1^ The attenuation of the peaks located between 1500 and 500 cm^–1^ in the film spectrum indicates the presence of electrostatic forces between the water and the polymer.

The formation of hydrogen bonds in film carrageenan is beneficial for e-tongue applications, since it might enhance the hydrogel’s ability to absorb and retain liquids, as it allows the polymer to effectively trap water within its structure, creating a stable and responsive sensing matrix. Corroborating with molecular analysis, the absorption capacity of the hydrogel was further confirmed by the contact angle measurements along with the determination of the time required for the hydrogel to complete absorb each liquid (Figure 2b), which provided detailed insights into the interaction between various liquids, such as coffee, water, milk, red and white wines, and the hydrophilic hydrogel surface. The corresponding contact angle images are depicted in Figure S2a–d. Coffee and milk exhibited the highest initial contact angles (∼42 and ∼39°), which suggests that the oils and organic molecules present in the beverages reduce their immediate interaction with the hydrogel’s surface, leading to lower wettability. Coffee and milk’s chemical composition includes hydrophobic compounds like lipids and certain organic acids, creating a barrier that delays liquid spreading and absorption. Despite this initial resistance, the hydrogel absorbed coffee and milk entirely within 17 and 16 s, respectively. This finding is significant, as it demonstrates the hydrogel’s ability to manage low-wettability liquids, overcoming the challenges posed by hydrophobic compounds.

Red and white wines show similar contact angles and absorption times. Red wine presents a contact angle of approximately 31° and takes about 13 s to be absorbed, whereas white wine presents a contact angle of 32° and an absorption time of 14 s. These samples show intermediate results of contact angle and absorption times compared with the other samples. On the one hand, the low pH and the presence of ethanol groups enhance the interaction between the wine and the hydrogel matrix by facilitating hydrogen bond formation. On the other hand, the presence of polyphenols, such as tannins, which exhibit a more hydrophobic behavior, contributes to the increase of the contact angle and the absorption times.

Water presents a low initial contact angle (∼18°) and short absorption times (8 s). This quick absorption highlights the hydrogel’s inherent hydrophilicity and strong affinity for polar molecules. Water’s simple molecular structure and high polarity allow it to easily penetrate and interact with hydrogel, facilitating fast absorption. The difference in absorption times between coffee and water reflects the hydrogel’s adaptive behavior toward liquids with varying chemical compositions. The hydrogel’s capacity to absorb all of the tested liquids within a short time frame, regardless of their initial contact angles, is indicative of its potential for broad-spectrum applications. This behavior suggests that the hydrogel can maintain consistent performance across different beverage types, ensuring reliable measurements and allowing the analysis of liquids with varying surface tension, viscosity, and chemical makeup without requiring modifications to the sensing material. Thus, the contact angle measurements and absorption times reveal rapid absorption of water and demonstrate strong hydrophilicity and adaptability.

**Figure 3:**
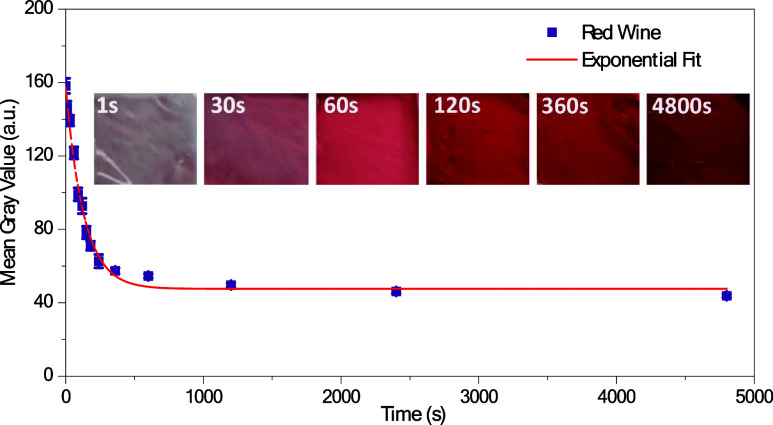
Colorimetric analysis of red wine absorption in the HITS system. Colorimetric calibration curves depicting the absorption of red wine samples over various timeframes, measured on the surface of the i-carrageenan hydrogel integrated into the HITS sensor. The results are presented as mean gray values with corresponding standard deviations obtained using ImageJ software. Representative photographs of the tested i-carrageenan hydrogels are also included, illustrating the visual changes in the hydrogel as red wine is absorbed. This analysis provides a visual and quantitative assessment of the sensor’s responsiveness to different liquid absorption dynamics.

Figure 3 presents the mean gray values of the carrageenan samples for different dipping times, relating the absorption dynamics of red wine by carrageenan hydrogels with absorption time through the colorimetric measurements. The inset of Figure 3 shows the photographic images of the samples before the mean gray value conversion. Photographs of the tested i-carrageenan samples are presented in detail in Figure S3. A pronounced decrease in mean gray values during the initial seconds of the absorption process is observed, indicating a swift phase of absorption, wherein the hydrogel absorbs a considerable volume of red wine. This initial phase is succeeded by a more gradual decline, which implies a deceleration in absorption as the hydrogel approaches saturation. This absorption behavior can be mathematically described by an exponential decay function, with an average residual error (reduced Chi-square) of 5.56. The equation used to find the experimental data in Figure 3 is as follows
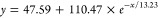
The inset images, captured at various intervals (1, 30, 60, 120, 360, and 4800 s), confirm the alteration in the coloration of the hydrogel as it assimilates red wine, which illustrates how the hydrogel’s chromatic properties evolve over time as a direct consequence of its interaction with red wine.

**Figure 4:**
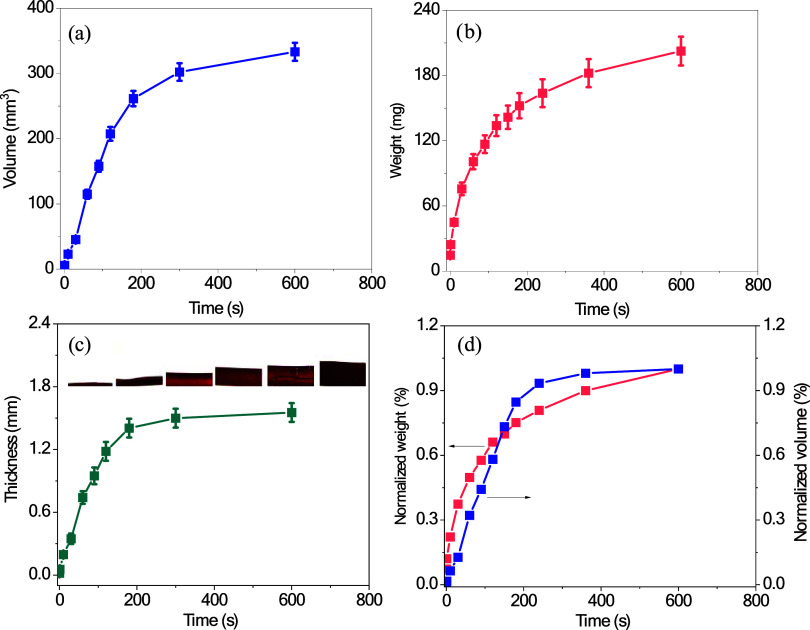
Swelling dynamics of i-carrageenan hydrogel in the HITS system. (a) Volume change of the i-carrageenan hydrogel sample as a function of time when exposed to red wine, measured in cubic millimeters (mm^3^), showing the expansion dynamics during absorption. (b) Weight change of the hydrogel over the absorption period, in milligrams (mg), reflecting the sample’s uptake of liquid. (c) Thickness variation of the hydrogel over time, in millimeters, with inset images capturing the visual progression of the swelling process, demonstrating the expansion of the hydrogel upon contact with the liquid. (d) Normalized weight and volume changes of the hydrogel over time, with the left *y*-axis representing the normalized percentage of weight change and the right *y*-axis representing the normalized percentage of volume change.

Figure 4a–c presents the variation of volume, weight, and thickness, respectively, of the i-carrageenan hydrogel over time during red wine absorption. An exponential volumetric increase is observed in Figure 4a, particularly within the first 180 s, where the volume surges from 1.80 to 261.51 mm^3^ (an increase of ≈14.31%), reaching approximately 350 mm^3^ by 600 s. Photographic images of the volume expansion for different absorption times are presented in Figure S4. Figure 4b depicts a similar weight variation trend. The hydrogel’s mass increases from 14.75 to 152.84 mg (an increase of ≈939.46%) in the first 180 s, before stabilizing near 210 mg at 600 s. The thickness variation, represented in Figure 4c, shows an analogous increase in thickness from 20 μm to 1.4 mm at 600 s, which reflects the hydrogel’s swelling during red wine absorption. The inset images visually confirm progressive swelling, demonstrating the inherent flexibility of the polymer network. A side view of the thickness of i-carrageenan hydrogel after 800 s is provided in Figure S2e, along with the interaction between the hydrogel and red wine after 10 s (Figure S2f). Figure 4d presents the normalized weight and volume changes over time, revealing a comparable trend for both parameters. The similarity between the normalized curves of weight and volume indicates that the structural expansion of the hydrogel is balanced between mass gain and volumetric change, confirming the uniform absorption of red wine. The underlying mechanism for this trend can be attributed to the hydrophilic characteristics of the i-carrageenan hydrogel, which is abundant in hydroxyl (−OH) and sulfate groups.^35^ These groups promote hydrogen bonding between the negatively charged sulfate groups in i-carrageenan and positively charged ions in red wine, such as potassium and calcium.

### Beverage Differentiation

The impedance spectroscopy results feature the effectiveness of the carbon interdigitated electrode in capturing distinct electrochemical signatures from different beverages. By analyzing the real and imaginary components of the impedance, shown in Figure S5, it was possible to distinguish between liquids, such as water, milk, red wine, white wine, and coffee, based on their unique chemical compositions (see the SI for principal components determination). The PCA employed in this study provided an effective method for simplifying and interpreting the complex impedance data gathered from the electronic tongue, allowing for meaningful insights into how different beverages interact with the sensor. By reducing the dimensionality of the data and generating new orthogonal components, PCA enabled the visualization of relationships between different beverages while retaining critical information from the original data set. The analysis was performed at a frequency of 1 kHz, chosen for its ability to minimize overlap between impedance response curves from different beverages and for the ease of use with simpler electronic readout circuits, thus enabling lower-cost instrumentation.

**Figure 5:**
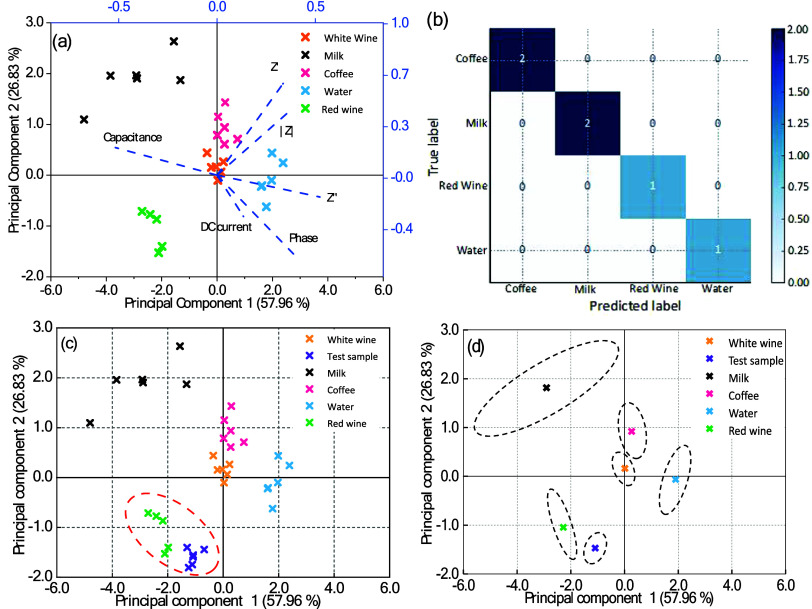
Data analysis for beverage classification using the HITS system. (a) PCA biplot showing the distribution of beverage samples based on impedance measurements, with vectors indicating the influence of each principal component on the classification of the samples. (b) Confusion matrix displaying the classification performance achieved by the HITS system, highlighting correctly classified instances on the diagonal and misclassified instances off-diagonal. (c) PCA score plot illustrating the clustering of different beverages according to their unique impedance signatures. (d) Scatter plot of centroids for each beverage class, accompanied by confidence ellipses to represent the variability and separation between the classes. The analysis was performed on data obtained from impedance measurements at 1 kHz, with a 15 mV amplitude signal, at a temperature of 25 °C. ML algorithms and AI techniques were applied to enhance the classification accuracy, as detailed in the Python programming scripts provided in the Supporting Information (SI). These scripts include data preprocessing, model training, and evaluation steps, underscoring the integration of advanced data analysis methods in optimizing the HITS system’s performance for reliable beverage recognition.

The PCA biplot in Figure 5a demonstrates that the first two principal components explained 84.78% of the total variation, revealing distinct clusters for each beverage sample. The clustering of replicates within each beverage type without overlapping between different types of beverages indicates the sensor’s high reproducibility and reliability.

Milk and red wine are clearly separated from other beverages due to their strongly negative PC1 values, while water stands out with its highly positive PC1 values. Coffee and white wine are more centralized in the PCA space with moderate variation along both dimensions. Along PC2, milk and coffee are positioned higher, while red wine occupies the lower region. The clear separation of the beverage clusters in the PCA plot highlights the electronic tongue’s ability to capture unique electrochemical signatures from each liquid, providing strong evidence of its discriminatory power.

The distinct chemical profiles of the tested beverages are influenced by variations in the pH levels, ionic concentrations, and organic compounds. These properties directly affect their electrochemical behaviors, which are effectively captured by the sensor system. Coffee, with its high levels of bitter compounds like caffeine and chlorogenic acids, along with oils, presented a distinct electrochemical signature (Figure S5). Its high initial contact angle reflects its lower wettability due to the hydrophobic nature of these oils, which resists interaction with the hydrophilic hydrogel. This property likely contributed to an increased charge transfer resistance (Rct) (Figure S5d) and a slower impedance response, which differentiated coffee from other beverages in the PCA plot. Despite this resistance, the electronic tongue successfully absorbed and identified coffee, showing its capability to handle more complex liquids with challenging electrochemical profiles. Milk, on the other hand, exhibited a different impedance response, primarily influenced by its moderate acidity (Table S1) and high content of organic molecules, such as proteins and lactose. These components likely increased the double-layer capacitance (Cdl; inset, Figure S5d) by forming a more substantial electric double layer at the electrode interface. This unique interaction resulted in a distinct cluster of milk samples in the PCA plot, highlighting the sensor’s ability to detect the influence of larger organic molecules on its electrochemical behavior.

The distinction between red and white wines is another example of the sensor’s sensitivity to compositional nuances. Red wine, with its higher polyphenol content due to the extended contact with grape skins during fermentation, demonstrated a higher charge transfer resistance (Rct) and an altered double-layer capacitance (Cdl) compared to white wine (Figure S5). It is possible that the interaction of tannins and alcohol with the electrode surface slowed electron transfer, creating a distinct impedance profile. White wine, with a lower polyphenol content but similar acidity, presented a different electrochemical response, reflected in a separate cluster in the PCA plot. The ability to differentiate red and white wines based on such subtle variations emphasizes the system’s effectiveness in distinguishing beverages with similar base compositions but different organic profiles. Water, serving as a neutral reference (Table S1), exhibited minimal impedance effects due to the lack of complex organic or ionic content. Its clustering in the PCA plot as a distinct, simple electrochemical profile confirmed the sensor’s ability to detect more complex variations in other liquids by providing a stable baseline for comparison.

### AI-ML-Based Blind Sensory Analysis

The use of blind sensory analysis aimed to differentiate various beverages using EIS combined with AI-ML. After collecting the EIS data from the different beverages, including red wine, white wine, coffee, milk, and water, an ML model was implemented to classify them based on their electrochemical signatures. PCA data were employed as a dimensionality reduction technique to focus on the most important features while retaining the essential electrochemical characteristics of each beverage.

The ML model was used to perform a single train–test split of the PCA data using 70% of the data for training and 30% of the data for testing (see the Machine Learning Details Section in the SI). After splitting, the data set was then classified using Random Forest algorithm with 100 estimators (number of decision trees) and random state of 42, to ensure the model’s reproducibility of results. Once trained, the model’s performance was evaluated using metrics such as accuracy, precision, recall, and F1-score (see Table S2). An accuracy of 100%, high precision, recall, and F1-scores were achieved for all beverage types. A confusion matrix, shown in Figure 5b, was also generated to visualize the model’s accuracy. Every entry in the diagonal corresponded to the correct number of classified samples for each beverage type, while the absence of nondiagonal entries showed no misclassifications. After the validation of the model, the electronic tongue system was subjected to a blind test using a red wine sample from a distinct brand not included in the training data set. For this test, a new i-carrageenan hydrogel sensor was prepared and used to measure another type of red wine. The same protocol for sensor preparation and EIS data collection was followed. Importantly, the machine was not informed that the sample was red wine, thereby eliminating any potential bias in the classification process.

The trained Random Forest model was used to make predictions about the EIS data collected from the blind sample and correctly classified the unknown sample as red wine, demonstrating its ability to generalize beyond the training data. The obtained PCA score plot calculated by the AI is shown in Figure 5c. The clear clustering of beverages in the PCA plot suggests that the system has significant potential for real-world applications, especially in the food and beverage industry, where distinguishing between products and ensuring quality control is critical. A data transformation of the PCA results was then performed to provide additional information about the distinctiveness and variability of the beverage categories based on the centroids, covariance, and variance for each beverage category. The transformation was done by calculating the mean of the PC1 and PC2 values for all samples in each category. The results are shown in Figure 5d, where each centroid represents the “center of mass” of the data points for each group and provides a simplified view of the group’s central location in the PCA-transformed space. To visualize the spread and variability within each beverage category, confidence ellipses were drawn around each centroid. These ellipses were calculated by using the covariance matrix of the PC1 and PC2 values for each category. The ellipses were drawn to represent 2 standard deviations from the centroid, which covers approximately 95% of the data points for each category. The centroid positions indicate that categories such as milk and water are well separated in the PCA space, with large distances between their centroids. The most notable observation comes from the two red wines whose centroids are positioned close to each other. The test sample centroid is at (−1.09, −1.59), while red wine is at (−2.27, −1.06). Both lie in the lower-left quadrant of the PCA plot, with negative values for both PC1 and PC2. This proximity suggests that the two categories share several characteristics, making them more challenging to distinguish from one another compared to other groups, such as milk or water. Although the test sample is less extreme in its PCA values, its centroid’s closeness to red wine indicates that there is a potential overlap between these two categories. The centroids highlight that milk, water, and coffee are well separated from the rest, suggesting easier classification of these categories. White wine and coffee have their centroids close, probably because of their similarities in terms of ionic and organic molecule (sugar, acid, and polyphenol) content, which can make the distinction between them challenging if other brands of the same products are added to the tests.

In terms of covariance and correlation, red wine shows a strong negative correlation between PC1 and PC2 (−0.80), meaning that the two components are inversely related in this group. Milk shows a high positive correlation (0.74), indicating that PC1 and PC2 increase together, reflecting the strong internal structure within the milk samples. Water has a moderately positive correlation (0.62), while white wine and coffee exhibit weaker correlations, meaning the principal components are more independent in these groups. The variance analysis highlights milk as the most variable group, with a variance of 1.76 along PC1, while white wine is the least variable group, with a variance of 0.04 on PC1.

The fact that the first two principal components explained nearly all of the variance also suggests that the system could operate with reduced data complexity, improving computational efficiency. This simplification allows for real-time analysis and classification of beverages, which are crucial for industries requiring rapid and accurate quality control. The ability to reduce data processing requirements without losing significant information is a major advantage for the practical deployment of the e-tongue in industrial settings. This result highlights the sensor’s sensitivity to the unique electrochemical properties of red wine, such as its specific charge transfer resistance Rct and Cdl, influenced by alcohol content and polyphenols. The absence of classification errors also indicates that the beverage types are sufficiently distinct within the PC1 and PC2 feature space, suggesting that the variability within each category is low with respect to these two dimensions. The scatter plot showing the correlation between predicted and true labels (see Figure S6) displayed a perfect correlation (1.00), with every true label matching its corresponding predicted label, as indicated by the fact that all data points lay exactly on the diagonal line of the plot.

In summary, the model effectively distinguishes between sample categories, demonstrating the electronic tongue system’s robustness for rapid and accurate beverage classification, especially in industries like food and beverage quality control. Though trained on a small data set, the model’s scalability suggests improved precision with larger data sets, offering the potential for broader applications, such as detecting counterfeit products or contaminants. Its autonomous, real-time processing reduces the need for human intervention, enhancing the efficiency and accuracy of sensory analysis processes while lowering labor costs in industrial settings. The HITS offers significant advantages compared to other electronic tongue technologies described in the literature. This system is distinguished by its high accuracy, achieving 100% success in classifying different beverages, combined with its rapid analysis time of only 5 min per sample. These features are crucial in applications requiring precise, efficient, and timely liquid analysis. Additionally, the use of eco-friendly materials, such as the biodegradable i-carrageenan hydrogel and recyclable PET substrate, underscores the environmental sustainability of the HITS system.

While other e-tongue systems may exhibit strengths in areas such as adaptability or specialization for certain applications, the HITS system stands out due to its unique combination of superior performance, reusability, and low environmental impact. This balance makes it a suitable option for a wide array of applications, including food and beverage quality control, health monitoring, and environmental sensing. Table 1 provides a detailed comparison of the HITS system with other electronic tongue technologies from the literature, highlighting its innovative contributions and practical advantages.

**Table 1:** Example of Different Electronic Tongue Technologies Applied in the Food Industry

sensors and materials	machine learning	beverage	key findings	accuracy	refs
carbon interdigitated electrodes with iota-carrageenan hydrogel on recyclable PET substrate	random forest, PCA	coffee, juice, water, white wine, red wine	sustainable, high-accuracy e-tongue system with rapid classification and low environmental impact.	∼100%; analysis time: 5 min/sample	this work
copolymeric hydrogel with zwitter-ionic and nonionic functional groups (DMAPS and HEMA)	machine learning	mixed beverages and foods	highly adaptive hydrogel-enabled sensing with high classification accuracy for diverse applications.	∼95%	(36)
saliva-ionic PAAm hydrogel with mucin and LiCl electrolytes	not specified	beverages and fruits	detects astringency with high sensitivity and fast response time.	∼90%	(37)
functionalized polymer-based sensors	deep learning	multiple flavor types	robust multitaste sensing using single-drop e-tongue design.	>90%	(38)
hydrogel with poly(vinyl alcohol) and benzene-1,4-diboronic acid	type-2 fuzzy classification algorithms	sugary beverages	high precision in identifying sugar types using hydrogel-enabled sensors.	∼97%	(39)
miniaturized NIR spectroscopy and ET sensors on polymeric substrates	extreme learning machines (ELM)	Black tea samples	effective black tea quality characterization using spectroscopy and e-tongue synergy.	∼93.56%	(40)

## Conclusions

This study demonstrates the potential of the HITS system as an innovative, sustainable, and highly effective solution for beverage recognition. By integrating a biodegradable iota-carrageenan hydrogel with a carbon-based interdigitated electrode on a recyclable PET substrate, the HITS system combines rapid and accurate sensing capabilities with environmental sustainability. The vibrational spectroscopic analysis revealed key structural transitions in the hydrogel that enhance its interaction with various liquids, while contact angle measurements and swelling dynamics confirmed its robust absorption properties across a range of beverage types. The use of impedance spectroscopy, analyzed with PCA and enhanced by machine learning algorithms, allowed the system to distinguish between beverages, such as milk, water, coffee, red wine, and white wine, with high accuracy, even in a blind sensory analysis. The ability to classify beverages based on unique electrochemical signatures underscores the versatility and reliability of the HITS system, making it suitable for real-time quality control and other industrial applications.

## Materials and Methods

i-carrageenan, a natural polysaccharide derived from red seaweed, was obtained from Alfa Aesar. Conductive carbon black ink, sourced from Ceres (Guangdong, China), was used to print the electrodes on a polyethylene terephthalate (PET) film, Dupont Teijin Melinex 506. Various beverages, including coffee, milk, red wine, and white wine, were sourced from local producers in Minho (Portugal) and commercially available brands. Deionized water, with a resistivity of 18 MΩ•cm at 25 °C, was prepared in the laboratory and used in all aqueous solutions.

### Electrode Preparation

The sensor’s platform was the carbon interdigitated electrode, fabricated using a manual screen printer (DSTAR, model DX-305D) equipped with a polyester screen mesh with 120 threads per centimeter. Conductive carbon black ink was used to print the electrodes onto a PET substrate. After the printing process, the electrodes were cured in an oven (JP Selecta, Model 2000208) at 80 °C for 20 min. The design included 10 fingers, each measuring 6 mm in length and 0.5 mm in width, with an interdigit space of 0.5 mm between adjacent fingers (Figure 1). This layout maximized the surface area for the interaction with the hydrogel layer, enhancing the sensitivity of the sensor.

### Hydrogel Preparation

To prepare the i-carrageenan hydrogel, 0.5 g of iota-carrageenan was dissolved in 16 mL of ultrapure water under constant magnetic stirring at 150 rpm for 3 h, ensuring the complete dissolution of the polymer. The solution was then poured into a Petri dish and allowed to rest for 72 h at room temperature to evaporate the solvent and form a thin hydrogel film. The final film exhibited a thickness of approximately 30 μm, which was ideal for forming a stable yet responsive layer on the electrode surface.

### Characterization Techniques

Fourier Transform Infrared (FTIR) spectroscopy was conducted by using a PerkinElmer UATR Two device to confirm the chemical composition of the i-carrageenan hydrogel in both powder and film forms. The spectra were collected over a range of 4000 to 400 cm^–1^ at room temperature, with 64 scans and a resolution of 4 cm^–1^. This analysis verified the functional groups present in the hydrogel.

The surface wettability of the hydrogel was assessed using the sessile drop technique conducted with a Data-Physics OCA20 instrument. Droplets (5 μL) of different liquids, including coffee, ultrapure water, milk, white wine, and red wine, were placed on the hydrogel surface, and the contact angles were measured. Six measurements were taken from different zones of the hydrogel for each liquid, and the average contact angle with standard deviation was reported. The time taken for the complete absorption of each droplet by the hydrogel was also measured, providing critical information on the hydrogel’s interaction with different liquid types.

Colorimetric analysis was conducted to monitor the absorption of red wine by the hydrogel over time. Images of the hydrogel surface were captured at intervals ranging from 1 to 4800 s using a smartphone camera. ImageJ software was used to calculate the mean gray values of the images, which were plotted over time to generate calibration curves, allowing for the quantitative assessment of color changes as the hydrogel absorbed the wine.

In addition to colorimetric analysis, the hydrogel’s physical changes in response to red wine absorption were also measured. The volume change of the hydrogel was tracked by measuring its dimensional expansion in cubic millimeters over a time range of 0 to 600 s. Simultaneously, the hydrogel’s weight was monitored using a Sartorius Cubis II Micro Lab precision balance, and the thickness of the hydrogel was measured in millimeters. Visual images of the swelling process were taken at different time intervals. Both the normalized weight and volume percentages were calculated and plotted to compare the absorption dynamics.

### Electronic Tongue System Application for Beverage Recognition through Impedance Spectroscopy

For beverage recognition using the HITS system, a 1 cm × 1 cm piece of i-carrageenan hydrogel film was cut from the original sheet and dip-coated into the beverage sample for 3 min. After absorption, the hydrogel was gently dabbed with paper to remove any excess liquid and then placed on the interdigitated carbon electrode of the HITS device (as shown in Figure 1). Impedance measurements were conducted at room temperature over a frequency range of 10 Hz to 10 kHz, with an AC voltage of 15 mV, using a PalmSens4 potentiostat. To ensure accuracy and reproducibility, each beverage was tested with five separate hydrogel samples, allowing the HITS system to provide reliable results. The impedance spectroscopy data, including parameters such as capacitance, impedance, and the real and imaginary components, were processed using PSTrace 5.9 and OriginLab software. These measurements were crucial for distinguishing the unique electrochemical signatures of the beverages, which were then analyzed using machine learning algorithms to enhance the classification performance. The HITS system’s ability to combine rapid analysis with advanced data processing highlights its potential for practical applications in beverage quality control.

### Data Analysis and Principal Component Analysis (PCA)

The impedance data from the electronic tongue were analyzed using principal component analysis (PCA). Data matrices were generated from the sensors’ responses, where rows represented each beverage sample and columns corresponded to the average impedance parameters. These matrices were then used for PCA, reducing the data size and enabling the identification of distinct beverage types. PCA was carried out using OriginPro 2024 software, which allowed for the effective visualization of how the beverages grouped based on their electrochemical signatures.

## Supporting Information Available

The experimental details, computational and ML details. **Experimental setup and data analysis**: Details on the preparation of iota-carrageenan hydrogel, sensor assembly, and data acquisition methods. **Machine learning models**: Description of PCA and Random Forest algorithms used for beverage classification, along with Python scripts. **Figures S1–S6**: Images illustrating sensor setup, contact angle measurements, red wine absorption, and impedance results. **Tables S1**–**S2**: pH levels of beverages and Random Forest evaluation metrics. **Additional Data**: Raw impedance data and step-by-step statistical analysis for reproducibility (PDF)
