# Supplementary material for: Hydrogel In-Tape Electronic Tongue
Source: ACS Appl Electron Mater. 2025 Feb 26;7(5):1792–801. doi: 10.1021/acsaelm.4c02059 (PMC11905930; doi:10.1021/acsaelm.4c02059)
Supplement: Supplementary file 1 — el4c02059_si_001.pdf [file el4c02059_si_001.pdf]

Supporting Information

Hydrogel In-Tape Electronic Tongue

Ricardo Brito-Pereira[‡a,b], Rita Policia[‡b,c], Clarisse Ribeiro[b], Pedro Martins[b], Senentxu Lanceros-Mendez[*a,b,d], Frank N. Crespilho[*b,e]

[a]BCMaterials, Basque Center for Materials, Applications and Nanostructures, UPV/EHU Science Park, 48940 Leioa, Spain
[b]CF-UM-UP, Centro de Física das Universidades do Minho e Porto, Universidade do Minho, Campus de Gualtar, 4710-057 Braga, Portugal
[c]IB-S, Institute of Science and Innovation for Bio-Sustainability, Universidade do Minho, Campus de Gualtar, 4710-057 Braga, Portugal
[d]IKERBASQUE, Basque Foundation for Science, 48009 Bilbao, Spain
[e]São Carlos Institute of Chemistry, University of São Paulo, São Carlos 13560-970, SP,Brazil

*frankcrespilho@iqsc.usp.br ; senentxu.lanceros@bcmaterials.net

Principal components determination

The data manipulation for PCA analysis was carried out according to the following steps:

1. Standardization of the data values was done according to the following equation, to pass all the variables to the same scale.

$$z = \frac{value - mean}{standart\ deviation}$$

2. Correlation Matrix Computation

The correlation matrix is done to understand how the variables of the input data set are related to each other.

	Phase	Current	|Z|	Z'	Z''	Capacitance
**Phase**	1	0.23028	0.15136	-0.07696	0.85045	-0.86886
**Current**	0.23028	1	0.04902	-2.44625E-4	0.20475	-0.2242
**|Z|**	0.15136	0.04902	1	0.97369	0.6474	-0.60628
**Z'**	-0.07696	-2.44625E-4	0.97369	1	0.45708	-0.41409
**Z''**	0.85045	0.20475	0.6474	0.45708	1	-0.98863
**Capacitance**	-0.86886	-0.2242	-0.60628	-0.41409	-0.98863	1

3. Computation of the eigenvectors and eigenvalues of the correlation matrix to identify the principal components.

Eigenvalues Matrix

	**Eigenvalue**	**Percentage of Variance**	**Cumulative**
**Phase**	3.47731	57.96%	57.96%
**Current**	1.60968	26.83%	84.78%
**|Z|**	0.90083	15.01%	99.80%
**Z’**	0.01167	0.19%	99.99%
**Z’’**	4.88E-4	0.01%	100.00%
**Capacitance**	2.1E-5	0.00%	100.00%

Eigen vectors matrix

	**Coefficients of PC1**	**Coefficients of PC2**
**Phase**	0.38842	-0.51982
**Current**	0.13437	-0.27299
**|Z|**	0.42166	0.48349
**Z'**	0.33644	0.60634
**Z''**	0.52282	-0.14396
**Capacitance**	-0.51647	0.18202

4. PCA biplot

In order to place the beverages in the PCA biplot (Figure 5a), the eigen vectors were plotted along with the score of each beverage. To calculate the scores, the transposed eigen vector matrix is multiplied by the standardized values of each sample.

ML Algorithms for Data Processing

The implementation of the artificial sensory analysis for beverage classification was developed using Python, leveraging the machine learning and data analysis libraries provided by the Python ecosystem. The core libraries employed include Pandas, Scikit-learn, NumPy, and Matplotlib, all of which are used for data processing, visualization, and model building (see Python Code Section). To process and classify the complex impedance data, machine learning algorithms were employed. The analysis was conducted in two key steps: dimensionality reduction using PCA and classification using the Random Forest algorithm. PCA was used to reduce the complexity, allowing the classification algorithm to focus on the most relevant features, making the subsequent analysis more computationally efficient and precise. After PCA, the Random Forest algorithm was used to classify the beverages based on their electrochemical properties. The algorithm was trained on the PCA-reduced dataset, where each beverage sample was labeled according to its type (e.g., milk, coffee, red wine, etc.).

To evaluate the robustness of the system, we conducted a blind test using a red wine sample from a different brand, not included in the training dataset. The goal was to assess whether the electronic tongue could accurately classify a beverage it had not encountered before. For this test, the i-carrageenan hydrogel sensor was prepared and exposed to the new red wine sample, following the same protocol as in previous experiments. EIS measurements were taken, and the resulting data were processed using the trained Random Forest model.

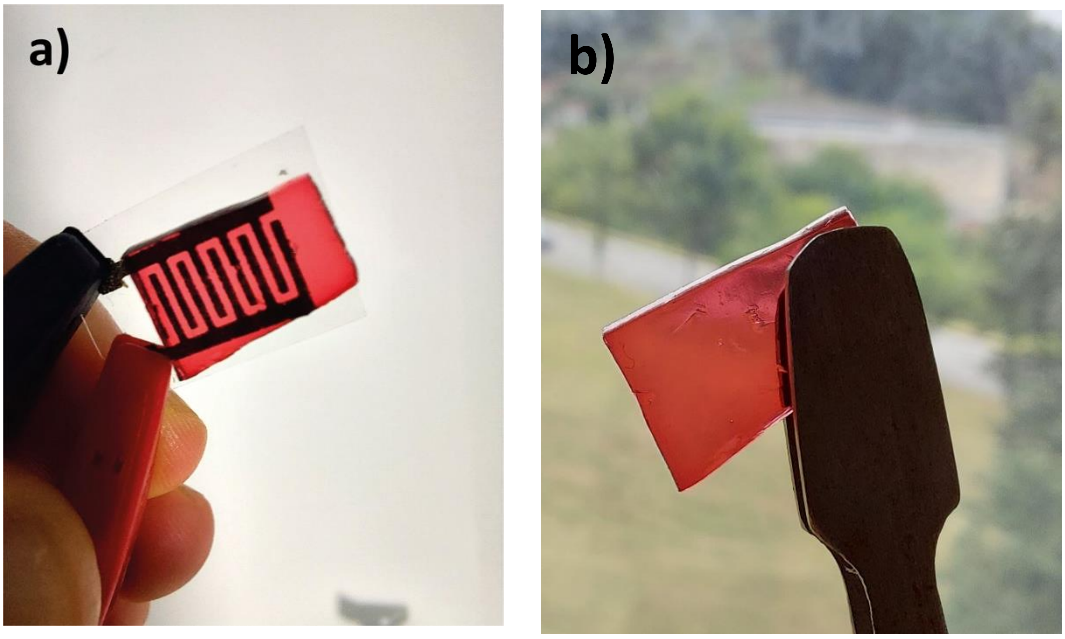

Figure S1. a) Image of the complete sensor connected to the potentiostat connectors b) I-carrageenan hydrogel after complete red wine absorption.

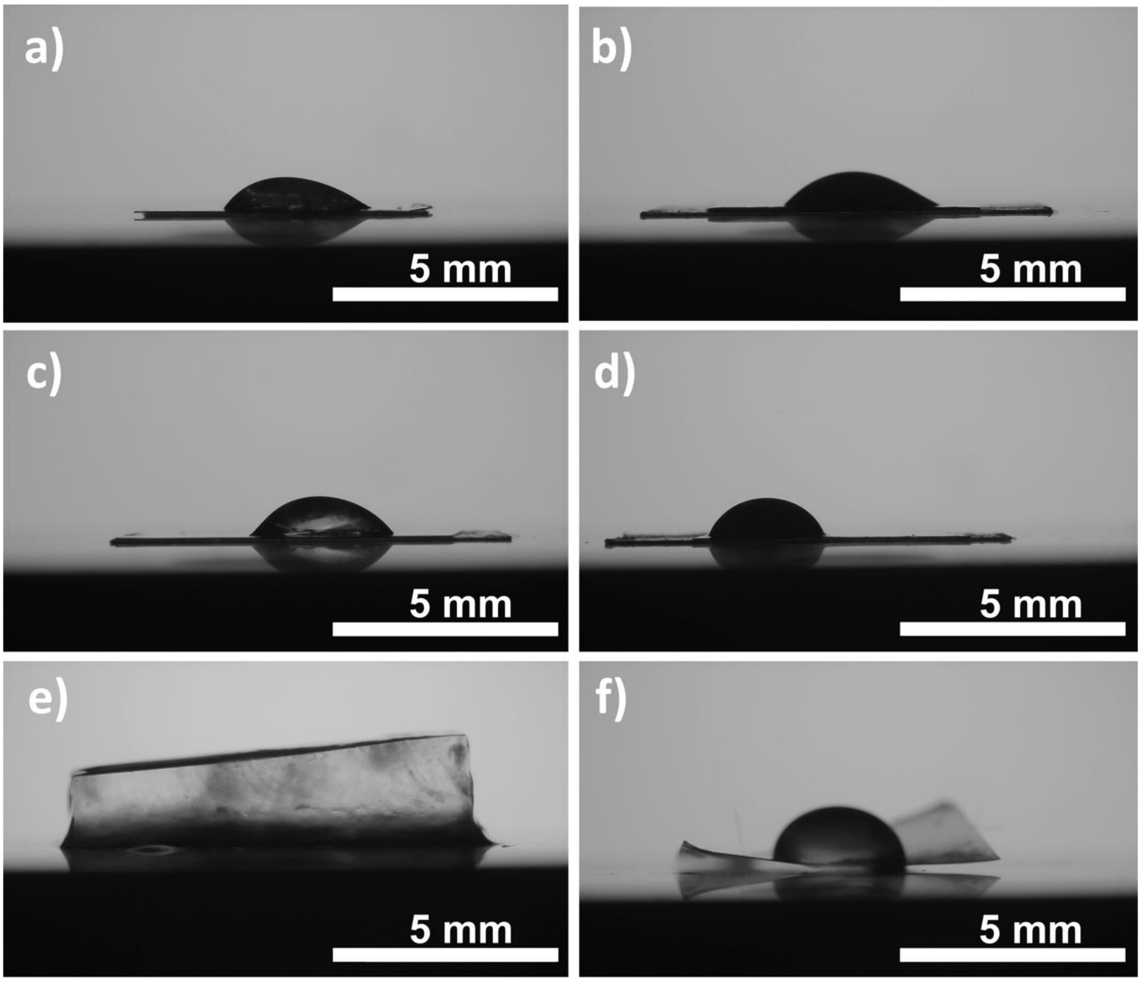

Figure S2. Contact angle images of a) Water; b) Red wine; c) White wine; and d) Coffee; e) Side view of the thickness of i-carrageenan hydrogel after dipping in red wine for 3 min; f) Interaction between i-carrageenan film and the liquid after 10s.

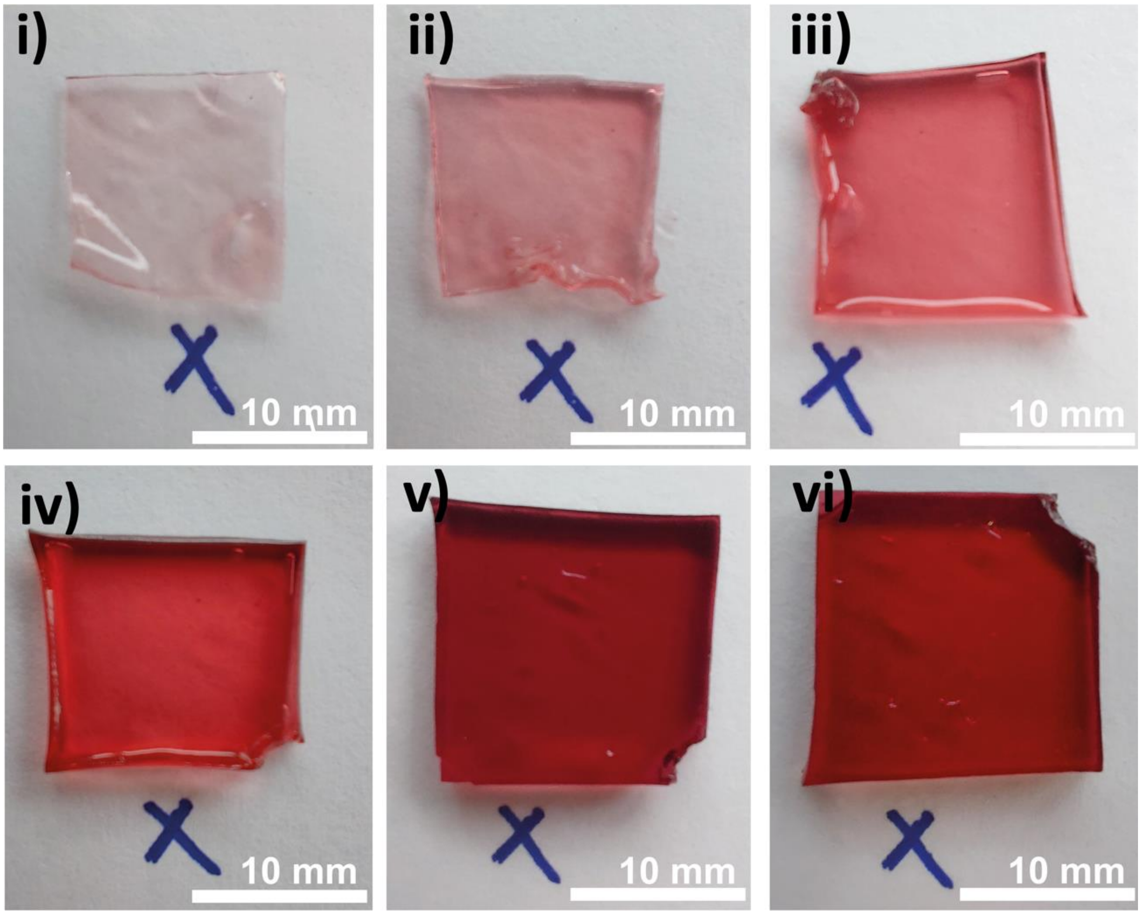

Figure S3. Representative photographs of the tested carrageenan hydrogels with different times of red wine absorption, i) 1s; ii) 30s; iii) 60s; iv) 120s; v) 360s; vi) 4800s.

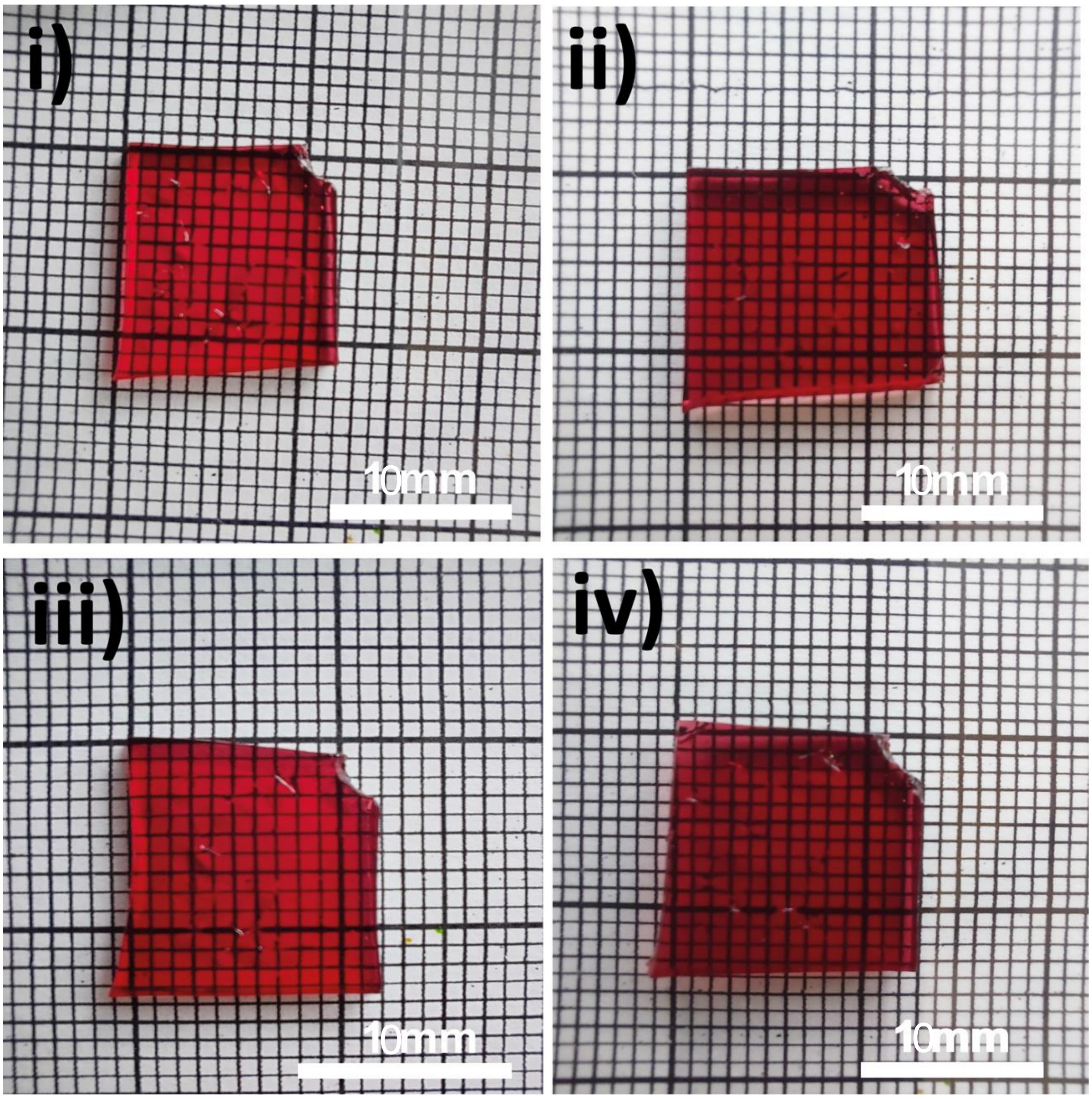

Figure S4. Volume change of the hydrogel sample as a function of time i) 60s; ii) 120s; iii) 180s; iv) 240s. All the samples are placed over millimetric paper, where each small square present dimensions of 1 mm x 1 mm.

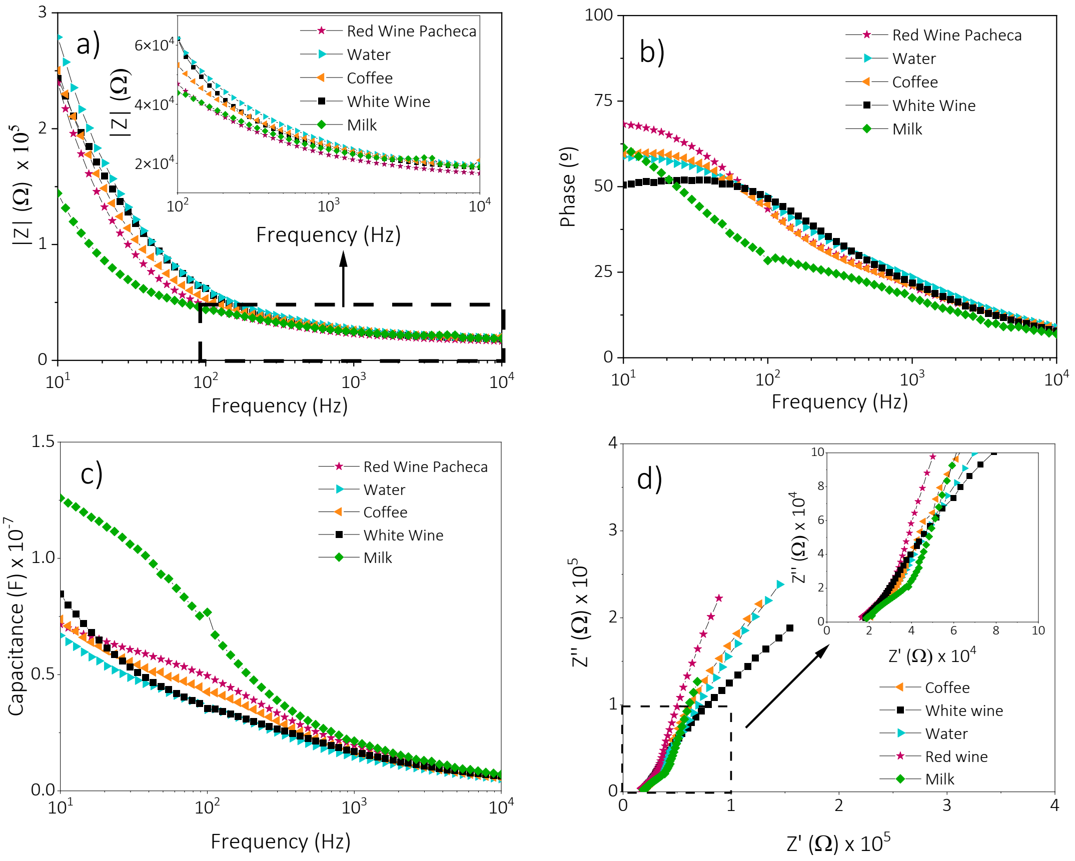

Figure S5. Impedance results. Plot of a) |Z|, b) phase, and c) capacitance as a function of applied frequency for interdigitated carbon electrodes with carrageenan films soaked in different drinks. Each curve represents the mean of five samples. AC amplitude: 15 mV at room temperature. d) Nyquist plot obtained from the impedance data for applied frequencies in the range of 10 – 1x104 Hz.

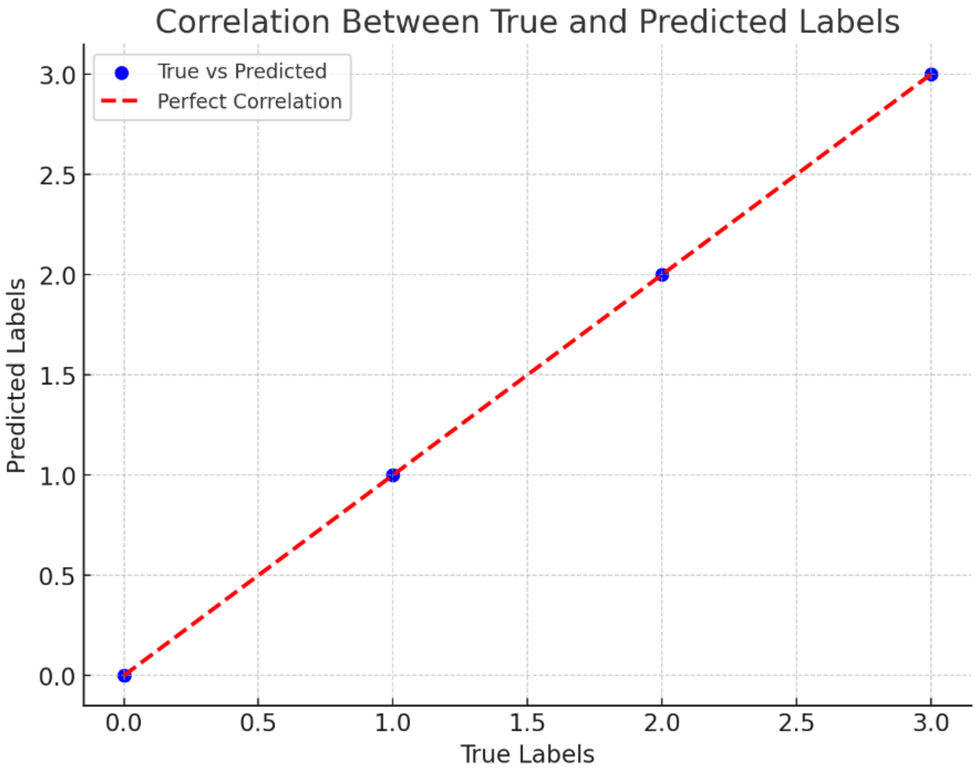

Figure S6. Correlation Between True and Predicted Labels.

Table S1. pH level of the different beverages used for e-tongue analysis.

Beverage sample	pH
**Milk**	6.4 – 6.8
**Coffee**	4.5 – 6.0
**Red Wine**	3.5 – 3.8
**White Wine**	3.0 – 3.4
**Water**	7.0

Detailed Machine Learning method

1. Data Preparation

The dataset was first prepared for use in a machine learning model. The PCA components (PC1 and PC2) were used as features, and the beverage types were the target labels. To enable the model to work with these categorical labels, the labels were transformed into numerical values using a class in scikit-learn named LabelEncoder. This allowed the model to interpret the different beverages as distinct categories.

2. Data Splitting

The dataset was divided into two parts:

Training Set: 70% of the data used to train the model.

Test Set: 30% of the data used to evaluate the model's performance.

3. Model Selection and Training

Random Forest algorithm was selected for classification. The number of decision trees was set to 100 and the random state was defined as 42, to ensure the model's reproducibility of results. The model was trained using the training data (PC1, PC2) and their corresponding beverage labels.

4. Model Evaluation

The trained Random Forest model was tested using the test dataset. It achieved a 100% accuracy rate, meaning it correctly classified all test samples. Afterwards, this model was evaluated through common evaluation metrics, namely precision, recall, and F1-score, as shown in Table S1. These metrics were calculated for each beverage category and showed perfect classification performance across all categories.

Table S2. Random Forest model evaluation metrics.

Beverage	Precision	Recall	F1-score
Coffee	1.00	1.00	1.00
Milk	1.00	1.00	1.00
Red Wine	1.00	1.00	1.00
Water	1.00	1.00	1.00

Given the perfect score across all metrics, we can infer that the model was able to distinguish between beverage types without error in this test case.

5. Unknown Sample Prediction

After training and validating the Random Forest model, we provided six samples of a different red wine for classification. The coordinates (PC1 and PC2) for these samples were:

[-1.11688, -1.74461],

[-1.30213, -1.40256],

[-1.08456, -1.55459],

[-1.24548, -1.80648],

[-0.68563, -1.44364],

[-1.08694, -1.59037]

The model classified all these samples as Red Wine. This classification aligns with the fact that the unknown samples' coordinates are in the same PCA region as previous Red Wine samples.

Python Code

# Importing libraries

```
import pandas as pd

import numpy as np

import matplotlib.pyplot as plt

import seaborn as sns
```

# Importing dataset

```
df = pd.read_csv('beverage_impedance_data.csv')  # Load the dataset

impedance_data = np.array(df.iloc[:, 1:])  # Convert relevant columns to NumPy arrays for processing
```

# Use of PCA to reduce from high-dimensional space (impedance results) to a two-dimensional space of 2 principal components

```
from sklearn.decomposition import PCA

pca = PCA(n_components=2)

principal_components = pca.fit_transform(impedance_data)

# Visualizing the results

plt.scatter(principal_components[:, 0], principal_components[:, 1], c=labels)

plt.xlabel('Principal Component 1')

plt.ylabel('Principal Component 2')

plt.show()
```

# Split the data into training and test sets

```
from sklearn.ensemble import RandomForestClassifier

from sklearn.model_selection import train_test_split

X_train, X_test, y_train, y_test = train_test_split(principal_components, labels, test_size=0.3, random_state=42)
```

# Initialize and train the Random Forest model on the PCA-reduced dataset to classify the beverages based on their electrochemical signatures

```
rf_model = RandomForestClassifier(n_estimators=100, random_state=42)

rf_model.fit(X_train, y_train)
```

# Predict and evaluate the model using the impedance data of the test sample

```
y_pred = rf_model.predict(X_test)

accuracy = np.mean(y_pred == y_test)

print(f"Accuracy: {accuracy}")
```

# Confusion Matrix

```
from sklearn.metrics import confusion_matrix

cm = confusion_matrix(y_test, y_pred)

sns.heatmap(cm, annot=True, fmt="d", cmap="Blues", xticklabels=beverages, yticklabels=beverages)

plt.ylabel('Actual')

plt.xlabel('Predicted')

plt.title('Confusion Matrix')

plt.show()
```
